# Patient-Specific Image-Based Computational Fluid Dynamics Analysis of Abdominal Aorta and Branches

**DOI:** 10.3390/jpm12091502

**Published:** 2022-09-14

**Authors:** Alin-Florin Totorean, Iuliana-Claudia Totorean, Sandor Ianos Bernad, Tiberiu Ciocan, Daniel Claudiu Malita, Dan Gaita, Elena Silvia Bernad

**Affiliations:** 1 Medical Engineering Group, Department of Mechanics and Strength of Materials, Politehnica University Timisoara, No 1 Mihai Viteazul Boulevard, 300222 Timisoara, Romania; 2 Cardiology Department, “Victor Babes” University of Medicine and Pharmacy, 2 Eftimie Murgu Square, 300041 Timisoara, Romania; 3 Institute of Cardiovascular Diseases Timisoara, 13A Gheorghe Adam Street, 300310 Timisoara, Romania; 4 Centre for Fundamental and Advanced Technical Research, Romanian Academy—Timisoara Branch, No 24 Mihai Viteazul Boulevard, 300223 Timisoara, Romania; 5 Research Center for Engineering of Systems with Complex Fluids, Politehnica University Timisoara, No 1 Mihai Viteazul Boulevard 1, 300222 Timisoara, Romania; 6 Department of Mechanical Machines, Equipment and Transportation, Politehnica University Timisoara, No 1 Mihai Viteazul Boulevard, 300222 Timisoara, Romania; 7 Department of Radiology, “Victor Babes” University of Medicine and Pharmacy, 2 Eftimie Murgu Square, 300041 Timisoara, Romania; 8 Department of Obstetrics and Gynecology, “Victor Babes” University of Medicine and Pharmacy, 2 Eftimie Murgu Square, 300041 Timisoara, Romania

**Keywords:** abdominal aorta, 3D reconstruction, patient-specific model, computational fluid dynamics, wall shear stress

## Abstract

The complicated abdominal aorta and its branches are a portion of the circulatory system prone to developing atherosclerotic plaque and aneurysms. These disorders are closely connected to the changing blood flow environment that the area’s complicated architecture produces (between celiac artery and iliac artery bifurcation); this phenomenon is widespread at arterial bifurcations. Based on computed tomography angiography (CTA) scans, this current work offers a numerical analysis of a patient-specific reconstruction of the abdominal aorta and its branches to identify and emphasize the most likely areas to develop atherosclerosis. The simulations were run following the heart cycle and under physiological settings. The wall shear stress (WSS), velocity field, and streamlines were examined. According to the findings, complex flow is primarily present at the location of arterial bifurcations, where abnormal flow patterns create recirculation zones with low and fluctuating WSS (<0.5 Pa), which are known to affect endothelial homeostasis and cause adverse vessel remodeling. The study provides a patient-specific hemodynamic analysis model, which couples in vivo CT imaging with in silico simulation under physiological circumstances. The study offers quantitative data on the range fluctuations of important hemodynamic parameters, such as WSS and recirculation region expansion, which are directly linked to the onset and progression of atherosclerosis. The findings could also help drug targeting at this vascular level by understanding blood flow patterns in the abdominal aorta and its branches.

## 1. Introduction

Computational fluid dynamics (CFD) has emerged in the past decade as a powerful tool in personalized medicine, enabling patient-specific modeling with multiple applications, particularly in the cardiovascular field [1]. CFD techniques are being used to simulate both standard and pathological conditions, improve diagnostic assessment, procedure planning, prediction of intervention outcome, and medical device optimization [1,2].

CFD offers tools to investigate cardiovascular physiology and compute several hemodynamic parameters that cannot be directly measured, for example, wall shear stress (WSS). The spectrum of applications in cardiovascular medicine is broad, ranging from coronary artery disease and physiology, stent design, study of aortic and cerebral aneurysms, and congenital heart disease investigation, to the evaluation and optimization of valve prosthesis and ventricular assist devices [2,3,4].

Several pathologies of the cardiovascular system, such as atherosclerotic plaque formation and aneurysm development, are strongly related to an altered blood flow environment [3,4]. It is well-established that changes in WSS lead to endothelial dysfunction and modify the behavior of smooth muscle cells. Complex flow dynamics are present mainly at the site of arterial bifurcations, where abnormal flow patterns lead to recirculation zones associated with low and oscillating WSS, which alter the endothelial homeostasis and induce adverse vessel remodeling through WSS-related signaling pathways and cellular response [4]. This continuous mechano-transduction process induces endothelial cell dysfunction, inflammation, flow stagnation, and smooth cellular proliferation (neointimal thickening), thus promoting atherosclerosis. CFD enables visualization and analysis of the hemodynamic changes underlying this complex phenomenon and helps to elucidate the links between flow environment and cardiovascular disease pathways [3,4,5,6].

The abdominal aorta and emerging branches are a section of the cardiovascular system that is highly susceptible to atherosclerosis due to its complex anatomy consisting of bifurcations and curvatures [7,8,9,10]. Moreover, the initialization and development of abdominal aortic aneurysms are also strongly related to the local hemodynamic microenvironment dominated by unsteady flow, complex flow, low WSS, and high oscillatory shear index [11,12,13]. Computational methods allow the investigation of flow patterns in vascular segments, thus providing critical insights into the pathophysiology and evolution of these pathologies. Furthermore, by accurately resolving flow in specific vascular geometries, these tools can identify the critical regions for atherosclerosis progression or aneurysm rupture [7,8,9,10,11,12,13].

CFD modeling coupled with cardiovascular imaging, either with computational tomography (CT) imaging or magnetic resonance imaging (MRI), provides patient-specific model reconstruction and non-invasive in silico simulations of flow within different areas of the cardiovascular system for both physiological and pathological conditions. Such image-based realistic models utilize the unique anatomy and physiology of each patient. They are used for individualized risk prediction and personalized procedures in virtual treatment planning [14,15,16,17,18].

The endothelium surface degraded markedly after being exposed to shear forces more significant than 350 to 400 dynes/cm^2^ for even an hour, according to early research by Fry [19]. Endothelial cytoplasmic enlargement, cell deformation, cell disintegration, and ultimately the breakdown and erosion of cell substance were all components of this degradation. Chronically exposing the endothelium surface of the artery to reduced shear pressures also caused subendothelial lipid deposition, substantial endothelial cell growth, and distortion of the nearby fibrillar architecture [20]. They postulated that low-shear regions adversely affected the bulk transport of lipids through the arterial wall because of Caro [21]. The occurrence of flow separation at branching points and bifurcations is one sort of hydraulic disturbance that promotes atherogenesis at these locations [22,23,24,25,26,27].

This study provides a complex CFD analysis of the hemodynamic parameters of a patient-specific abdominal aorta and branches geometry reconstruction, based on computed tomography angiography (CTA) images, to identify and emphasize the most likely areas to develop atherosclerosis. The hemodynamic analysis is performed following the cardiac cycle and under physiological settings, providing valuable insights into the complexity of blood flow patterns for this specific area of the cardiovascular system. In addition, quantitative data is provided on the range fluctuations of important hemodynamic parameters, such as WSS and recirculation region expansion, which are directly linked to the onset and progression of atherosclerosis [21].

## 2. Materials and Methods

### 2.1. Image Acquisition

The study is based on a 51-year-old female clinical dataset. The study was conducted following the Declaration of Helsinki and approved by the Ethics Committee of the Emergency Municipal Hospital Timisoara (No. I-18470/15.07.2022). The 51-year-old female patient was admitted to the hospital for routine testing since she had cardiovascular risk factors (age, hypertension, diabetes, dyslipidemia). In addition, the patient’s abdominal region was subjected to a CTA imaging study as part of these investigations.

Computed tomography angiography scans were performed on a 16-slice scanner (GE Healthcare, Brightspeed, USA). A 20 mL contrast agent (Omnipaque, Iohexol 350 mg/mL, GE Healthcare, Oslo, Norway) was injected intravenously at a flow rate of 4 mL/s. In addition, 100 mL of contrast agent was administered. The scan started automatically when a threshold of 144 HU (Hounsfield units) [28] was reached in a region of interest positioned in the superior abdominal aorta. The detector collimation was 1.25 mm, gantry rotation speed was 0.8 s per rotation, and tube voltage was 120 kV at a current of 85 to 183 mA. Scanning coverage was 235.1 mm with 35 mm/rot table feed. The reconstruction was performed with a standard convolutional kernel configuration. Thresholding and seeded region growing techniques were subsequently applied to segment the renal arteries and the aorta. The 3D model segmentation was based on reconstructed images with a slice thickness of 1.25 mm.

### 2.2. Geometry Reconstruction

**Figure 1:**
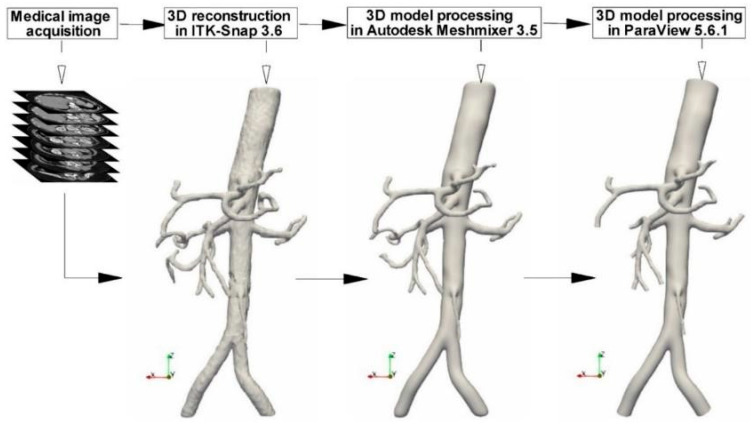
3D model reconstruction based on the DICOM images acquired during the CTA investigation.

Based on the CTA files, a 3D model of the abdominal aorta and its developing branches were created. Several open-source software programs are used in the pipeline to reconstruct the image-based model and set it up for numerical analysis, as depicted in Figure 1.

**Figure 2:**
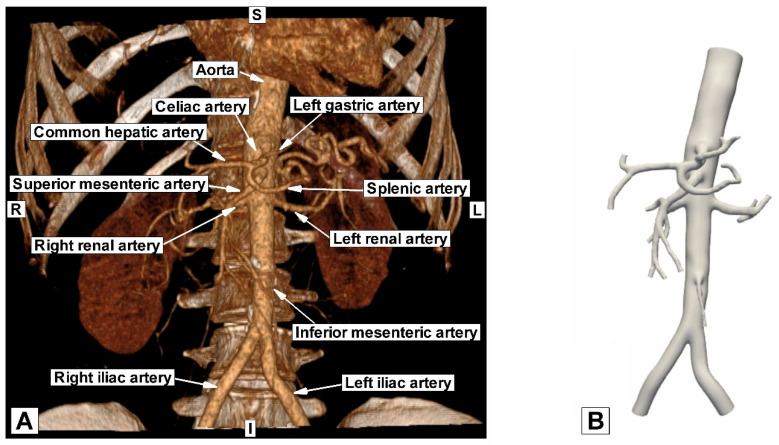
Comparison between geometry reconstruction using (**A**) 3D VRT (volume rendered technique) based on CTA images; and (**B**) advanced numerical techniques.

Shortly, the initial phase was importing the DICOM (Digital Imaging and Communications in Medicine) images into ITK-Snap 3.6.0. First, the 3D model was created using manual and automatic segmentation while considering the anatomical characteristics. Second, the model’s “stl” file was exported, then imported into Autodesk Meshmixer 3.5 for remeshing and surface smoothing. Third, to create flat inlet and outlet sections in ParaView 5.6.1, the arterial model was clipped in the input and output regions using planes perpendicular to the arteries’ centerlines. Finally, the clipping procedure was used to create the entrance and outlet faces’ normal vectors parallel to the direction of blood flow. The previous three steps must be completed in about 10 h for an aorta geometry like the one in Figure 2 to be successful.

**Figure 3:**
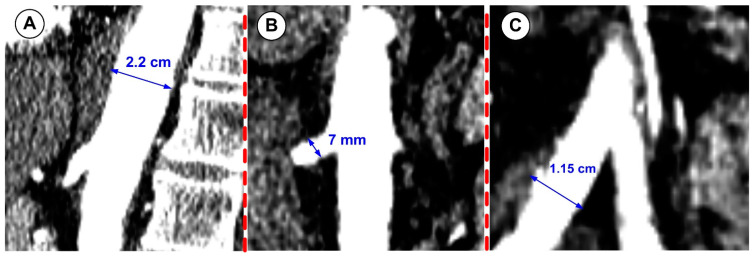
Abdominal aorta branching arteries’ diameter measurements during image acquisition. (**A**) abdominal aorta; (**B**) right renal artery; (**C**) right iliac artery.

Figure 2B displays the reconstructed patient-specific abdominal aorta and the developing branches based on the CTA image presented in Figure 2A. Table 1 shows the geometric parameters of the reconstructed patient-specific abdominal aorta and comparison with values presented in the literature for the same arterial segments; Figure 3 presents the diameter measurements of the arteries during geometry reconstruction.

### 2.3. Geometry Meshing Method

**Figure 4:**
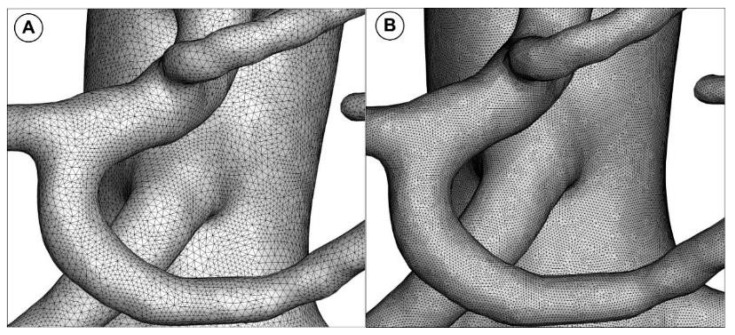
Unstructured volumetric mesh generated for the arterial model numerical investigation. (**A**) coarse mesh (2,885,022 elements); (**B**) fine mesh (9,558,980 elements).

The commercial tool ANSYS^®^ Mesher (ANSYS, Canonsburg, PA, USA) was used to create the computational mesh. The reconstructed abdominal aorta geometry was discretized using volume meshing with tetrahedral elements. For the final numerical domain analysis, about 9,500,000 tetrahedral elements with a boundary layer made of prism elements next to the wall were used (Figure 4). For the analyzed aorta geometry, the size of the elements for the final mesh ranges from 0.25 to 0.5 mm.

**Figure 5:**
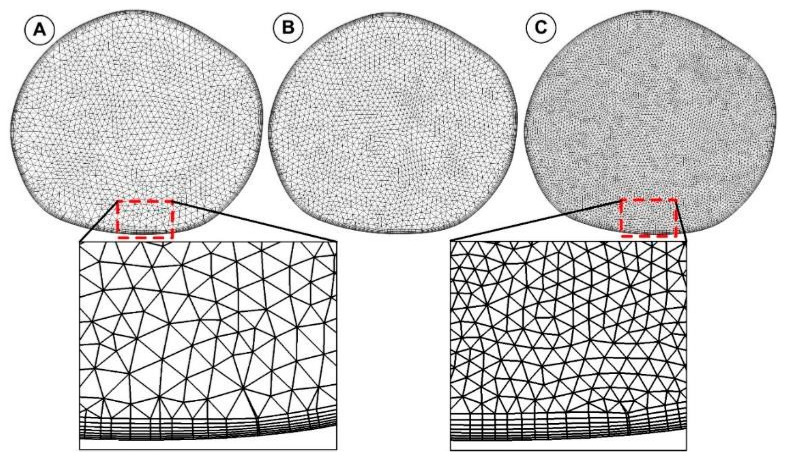
Cros-sectional view of the mesh topology and the generated boundary layer. The figure shows the mesh generated for the inlet section. (**A**) coarse mesh (4582 cells); (**B**) medium mesh (6386 cells); and (**C**) fine mesh (13,782 cells).

To capture the flow close to the wall, a prism layer comprised of seven layers with a height ratio of 1.2 and a first layer thickness of 0.025 mm was used (Figure 5). As the turbulent transition model required, the cell’s dimensionless height (y+) next to the wall in the current work was kept below 2 (y+ = 1).

The total elements and nodes used for the mesh abdominal aorta and the branches were 9,558,980 and 3,479,629, respectively. Different internal parameters assessed the quality of the meshes. For example, the average skewness is 0.1982 (maximum of 0.950 and minimum of 2.858 × 10^−4^), even in the most tortuous area, which was entirely satisfactory. This mesh of domains is used for computational analysis.

### 2.4. Computational Fluid Dynamics

**Figure 6:**
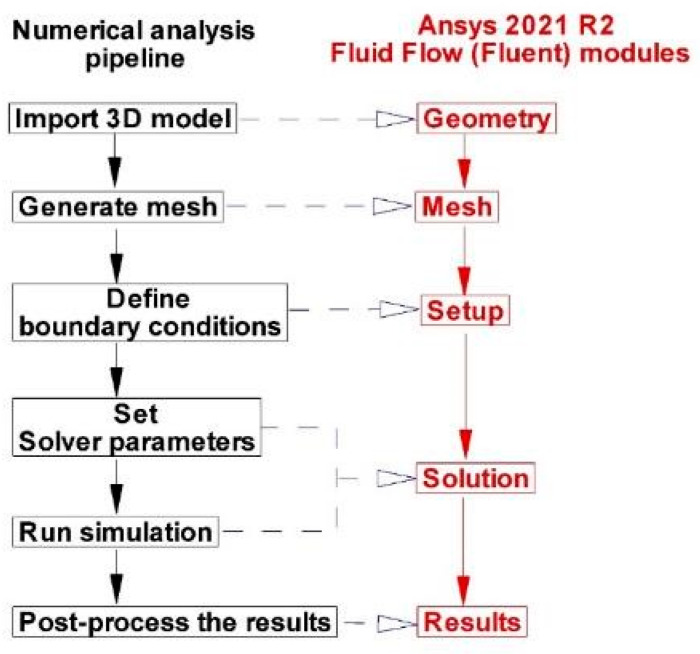
Pipeline for numerical analysis.

This paper’s numerical analysis approximates the Navier–Stokes equations’ solution by simulating the fluid’s motion [26,27]. This type of analysis makes it possible to obtain a thorough description of the flow variables (such as pressure, velocity, and wall shear stress) as functions of both space and time throughout the entire fluid domain [31], and it can result in accurate quantitative analyses and predictions of fluid flow phenomena. A schematic representation of the numerical simulation method gives the approach an overview in Figure 6.

The Navier–Stokes equation [32] governs blood flow in the vascular system. The finite-volume computational fluid dynamics algorithm Ansys-Fluent (Ansys 2021, Ansys Inc., Canonsburg, PA, USA) [33] was used to accomplish this. The velocity-pressure correction was performed using the SIMPLEC algorithm, a 3D single-precision format, and a segregated solver. The standard format was adopted for the pressure discretization and the second-order upwind format for the momentum equations. The continuity equation based on the mass conservation principle and the momentum equation based on the momentum conservation principle (Newton’s second law) is the ensemble-averaged governing equations that have been solved for mass and momentum conservation.

The blood flow is assumed to be irregular, isothermal, and incompressible, with a specific mass density of 1060 kg/m^3^ and dynamic viscosity of 0.0036 Pa.s. The irregular heart cycle is only a one-time point in the steady simulations’ flow circumstances (used for mesh independence analysis). This period produces a Reynolds number based on the vessel diameter, Re = 3129, and the maximal flow rate during systole.

Most major arteries show a change in artery width during the cardiac cycle that is less than 10% under typical resting conditions [34]. As a result, the vessel walls were rigid, and the no-slip wall boundary condition was applied [30,32].

Each simulation began with a velocity and pressure of zero and was then carried out using a segregate solver. The time step for all simulations was 1 × 10^−3^ s (20 iterations for each time step). Each simulation ran roughly 2.5 days to complete and was run on 16 cores on a Dell 32-core server with 128 GB of RAM.

### 2.5. Turbulence Model

Flow areas distant from the artery bifurcation and the 3D curved artery segment are characterized by transitional and turbulent flow characteristics that can produce moderate to severe arterial stenosis. This flow is not suited for laminar flow modeling, but the Reynolds-averaged Navier–Stokes (RANS) based turbulence models are designed for totally turbulent flow investigations [23,35,36,37,38]. Therefore, the transitional shear stress transport (SST) k-ω model, developed to represent the transition to a turbulent state, is used in this study [39,40]. The transitional model solves the SST k-ω transfer equations in terms of momentum-thickness Reynolds number using two additional equations, one for intermittency and one for transition initiation conditions. In addition, the model uses an empirical correlation-based approach to account for the influence of pressure gradient, external turbulence level, and transitional length on turbulent transition and local flow characteristics [39].

### 2.6. Boundary Conditions

**Figure 7:**
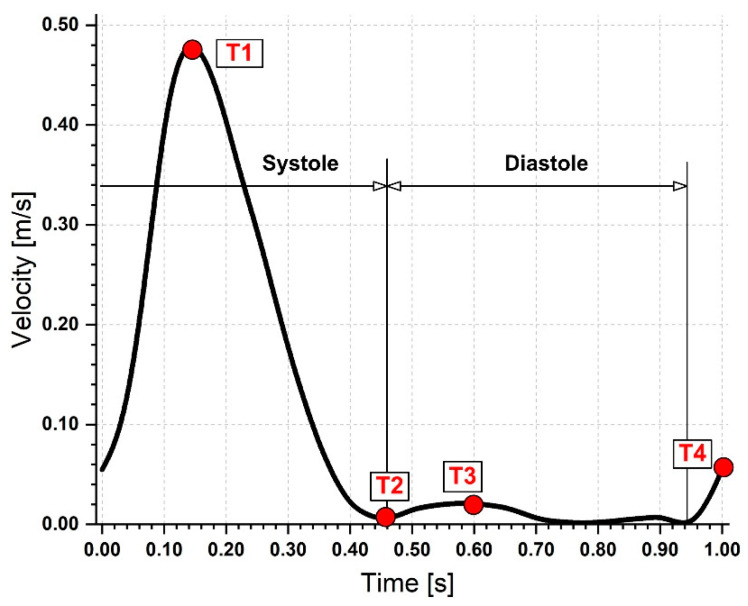
Physiologically realistic velocity waveform used in the numerical simulation. Time steps used for hemodynamic parameters investigation.

The inlet boundary condition was considered a pulsatile inlet velocity waveform (plug profile) with a cardiac period of 1 s from Liu [41], and there was no secondary flow at the inlet. Figure 7 shows the waveform of velocity against time.

Four cardiac cycles were simulated for each transient simulation to guarantee statistical convergence or periodicity. However, it was noted that the results exhibited identical periodicity following the first cardiac cycle. Therefore, we examined the information from the second cardiac cycle to report the findings. In addition, the cardiac cycle’s wall shear stress, pressure, and velocity contours were examined. The Reynolds number, Re_D_, based on the intake aortic diameter D, was around 3100 at peak systole with a Womersley number of 46. Since Re_D_ was higher than the critical Reynolds number for transition to turbulent flow, this indicates that we have a transient flow regime.

The Womersley number defines the velocity profile applied at the inlet surface. Values closer to 1 are associated with parabolic profiles, while higher values are associated with uniform profiles. Since the ostium surface is not a perfect circle, the Womersley number for each case was calculated considering the radius of an equivalent circle with the same area.

The pressure outlet condition was applied; specifically, the entire artery exit sections had a pressure of 0 Pa. Ansys-FLUENT modifies the gauge pressure field after each iteration for incompressible flows without pressure boundaries to prevent it from floating. The pressure in the cell closest to (or at) the reference pressure position is used for this. Simply using the outlets as a reference site for zero pressure is sufficient to get reliable findings because, in our work, the entire outlet sections are roughly symmetric [42].

**Figure 8:**
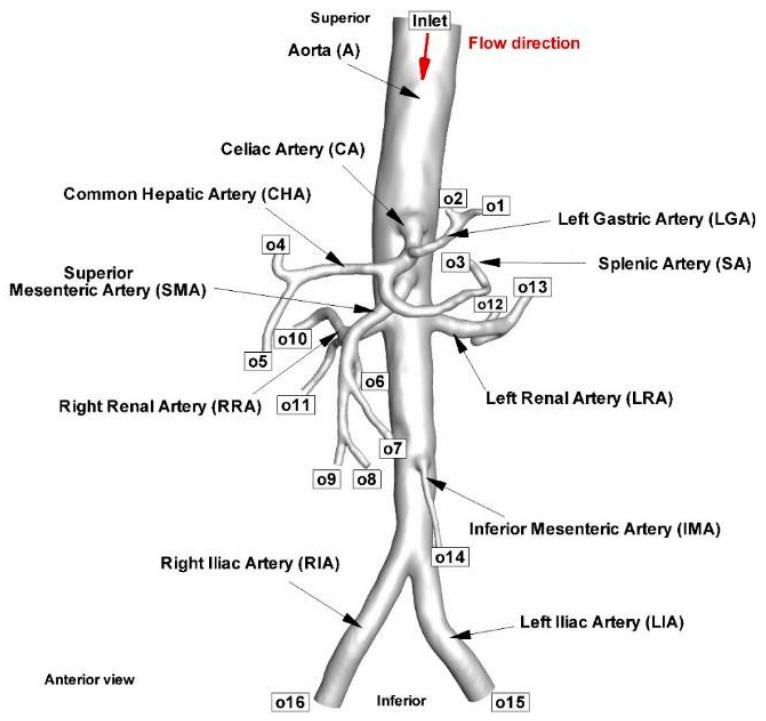
Anatomical representation of the model of the examined blood vessels, including the iliac arteries, major abdominal arteries, and their relationship to the abdominal aorta. Abbreviation for each investigated arterial branch and arterial segment.

The inlet and outlet surface portions of the artery geometries were assumed to have typical flow entry and exit from the calculation domain (Figure 8).

Blood was modeled as a Newtonian fluid in this study. For example, [43,44,45] shows that the average difference in WSS between Newtonian and non-Newtonian models is 10%.

The time step size considered in the fluid domain was 0.001 s each time step, the number of iterations for each time step was 20, and the time step number defined was 1000, which corresponded to the period of four cardiac cycles (4 cardiac cycles at 1 s each = 4 s).

For the residuals of the continuity equation and the X, Y, and Z momentum equations, the convergence criteria were set to 10^−6^.

### 2.7. Grid Independence Study

A grid independence analysis was performed to guarantee the independence of the results to the mesh. The sensitivity analysis was performed for steady-state flow condition (v_inlet_ =0.25 m/s, Q = 5.64 L/min, Re = 3123). Three computational meshes of 2.8 M, 6.5 M, and 9.5 M cells were tested (Figure 3). Table 2 displays the numbers of nodes and elements created in three different mesh configurations.

## 3. Results

A comparison of numerical results showed that the relative difference in terms of peak velocity and flow rate at the boundaries were less than 0.83% and 0.8%, respectively, between the coarse and medium mesh, and less than 1.13%, and 1.11%, respectively, between the medium and fine mesh. Therefore, we chose the fine mesh with 9.5 M elements to perform the numerical analysis to ensure a correct spatial representation of the hemodynamic parameters.

**Figure 9:**
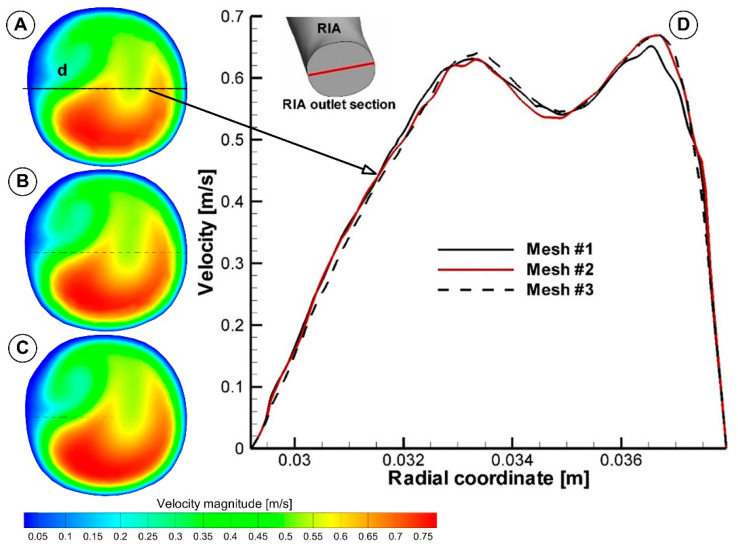
Mesh sensitivity test for the velocity profile associated with the right iliac artery (RIA) diameter in the outlet section (**D)**. “d” diameter of the exit section of RIA. (**A**) coarse mesh (mesh#1); (**B**) medium mesh (mesh#2), and (**C**) fine mesh (mesh#3). (**A**–**C**) shows the velocity magnitude contour in the outlet section of the RIA.

The mesh sensitivity test for the aorta model was also tested for the velocity profile evolution at the diameter of the outlet section of the right iliac artery (RIA) (Figure 9). The difference in maximum velocity value between fine and medium mesh was 0.09%, but between medium and coarse mesh was 3%.

Four separate time points were used throughout the cardiac cycle to measure the hemodynamic parameters. The essential flow rate stages of the cardiac cycle are represented by these four-time instants: T1 = 0.15 s (peak systole), T2 = 0.45 s (maximum deceleration), T3 = 0.6 s (maximum diastole), and T4 = 1 s (mid-acceleration). The average flow rate distribution between each branch and the overall flow rate was calculated. About 75% of the blood goes through the iliac artery bifurcation, and the remaining 25% is spread among the abdominal branches (Table 3).

### 3.1. Velocity Field and Profiles

**Figure 10:**
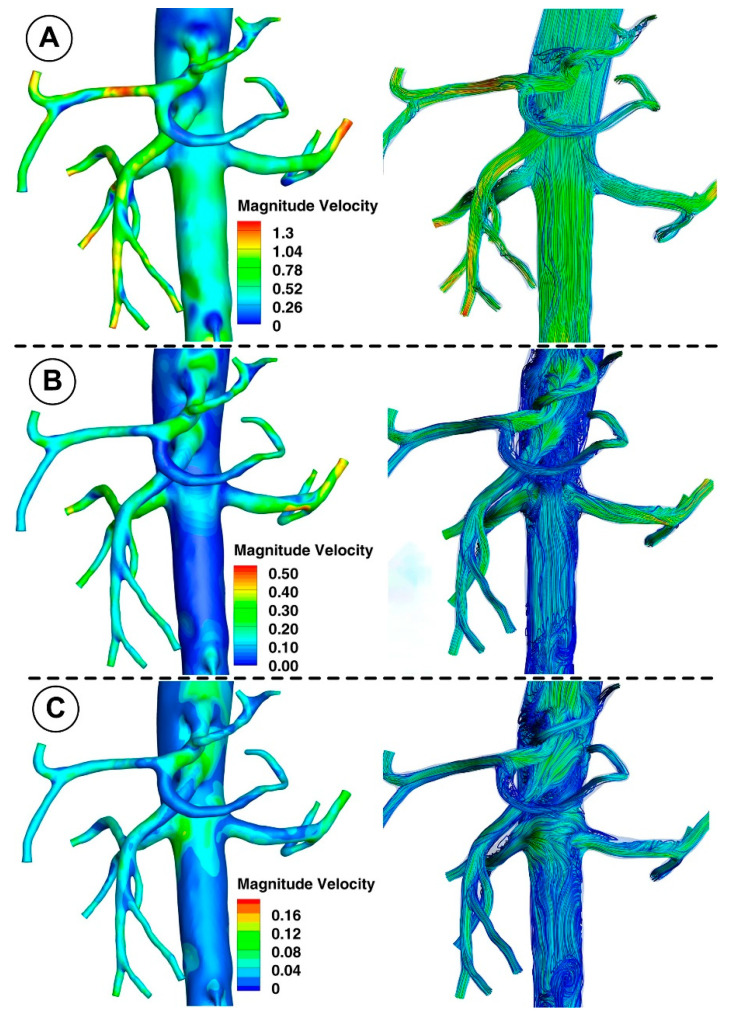
Representative 3D image of a reconstructed artery’s bifurcation segment showing the distribution of WSS and the path line for different time steps corresponding to the investigated cardiac cycle. (**A**) T1 = 0.15 s; (**B**) T2 = 0.45 s; (**C**) T3 = 0.6 s.

**Figure 11:**
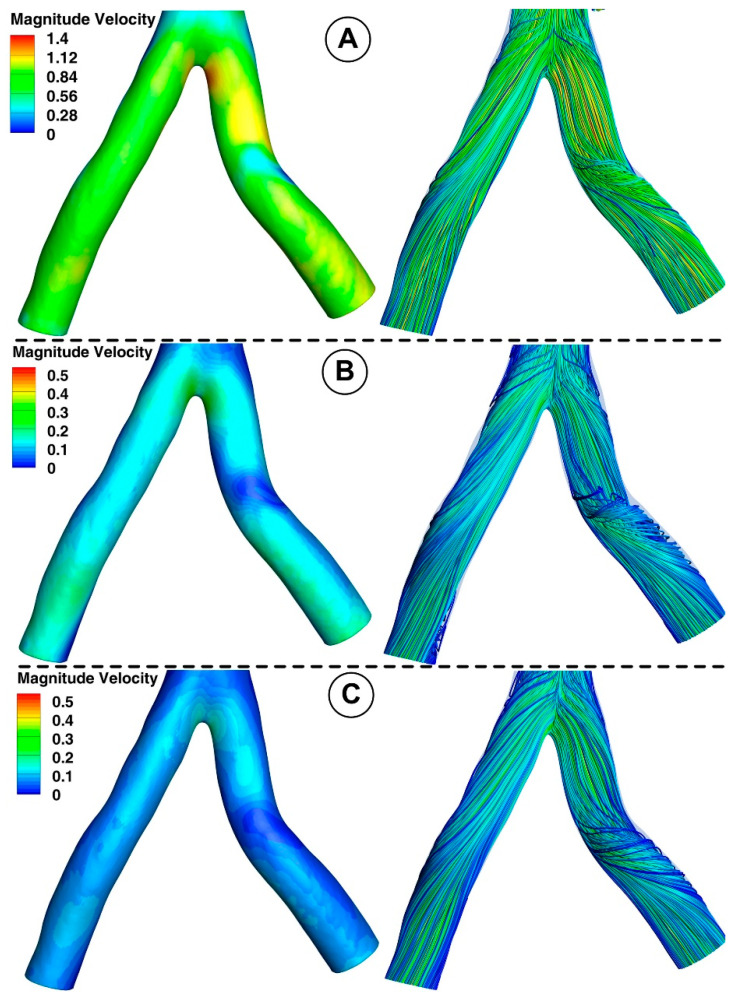
Velocity field distribution (**left**) and the path line (**right**) in the iliac artery bifurcation for different time steps. (**A**) T1 = 0.15 s; (**B**) T2 = 0.45 s; (**C**) T3 = 0.6 s.

Figure 10 and Figure 11 show the velocity fields in the abdominal aorta derived from numerical simulations. The findings demonstrate the flow evolution throughout a cardiac cycle. During the systolic and maximum-deceleration phases, highly organized motion is seen (Figure 10A,B), reaching maximum velocities of 1.4 m/s at the abdominal aortic wall. On the other hand, disorganized streamlines characterize the flow around the ostium of the branches during the maximum-diastole phase, with velocity values up to 0.4 m/s around the aortorenal branch (Figure 10) and 1.2 m/s in the iliac artery bifurcation region (Figure 11), respectively. As can be seen in Figure 10A,B, the flow separation region at the inner curvature of the branch forms during the systolic and deceleration phase. The 3D streamlines produced by the computational model demonstrate the 3-dimensional aspect of the flow (Figure 10; Figure 11, right column). Figure 10A and Figure 11A show ordered streamlines during the systolic phase in the abdominal aorta, where fluid flows present parallel layers and disordered 3D flow is visible during the maximum-deceleration and diastolic phases (Figure 10B,C and Figure 11B,C, respectively).

According to clinical findings, plaques predominately form in areas where the flow is locally interrupted and there are high levels of wall shear stress [46]. In addition to geometry, numerous parameters significantly impact the flow pattern [47,48,49]. They include the Reynolds number (flow rate), branching ratio, and waveform. The vascular system’s blood flows are irregular; the aorta’s flow rate constantly fluctuates, causing the blood flow to accelerate and decelerate.

According to fluid dynamics principles, flow separation happens when the fluid’s forward inertia cannot withstand the pressure gradient acting against it. Therefore, flow reversal occurs when the blood flow along the aorta is in the deceleration phase, complicating the flow pattern in the aorta, especially in arterial bifurcations.

A bifurcated iliac artery has geometric implications that result in significant flow skewness and recirculation. The generation of boundary layers after the bifurcation point, which results in flow skewness toward the inside wall of the junction and the creation of secondary motions, has been shown in several flow visualization investigations of bifurcated geometries [50].

This investigation identified that the geometrical impact of the out-of-plane artery model was the primary influencing element. Figure 11 demonstrates how the flow moved in the iliac artery branches in a helical pattern. As shown, the iliac artery bifurcation causes the branches to experience a helical form of flow. So, while the LIA has a right-handed helical flow, the RIA has a left-handed helical flow. The relationship between hemodynamic parameters (velocity field and flow direction) in various regions of the iliac artery where it divides into two is also depicted in Figure 11.

### 3.2. Wall Shear Stress

The branches of the abdominal aorta found in living things have numerous bends and twists. Due to the centrifugal force acting on the blood flow, WSS tends to be high on the outer radius and low on the inner radius at every bend, significantly increasing the maximum WSS from that of a straight section.

**Figure 12:**
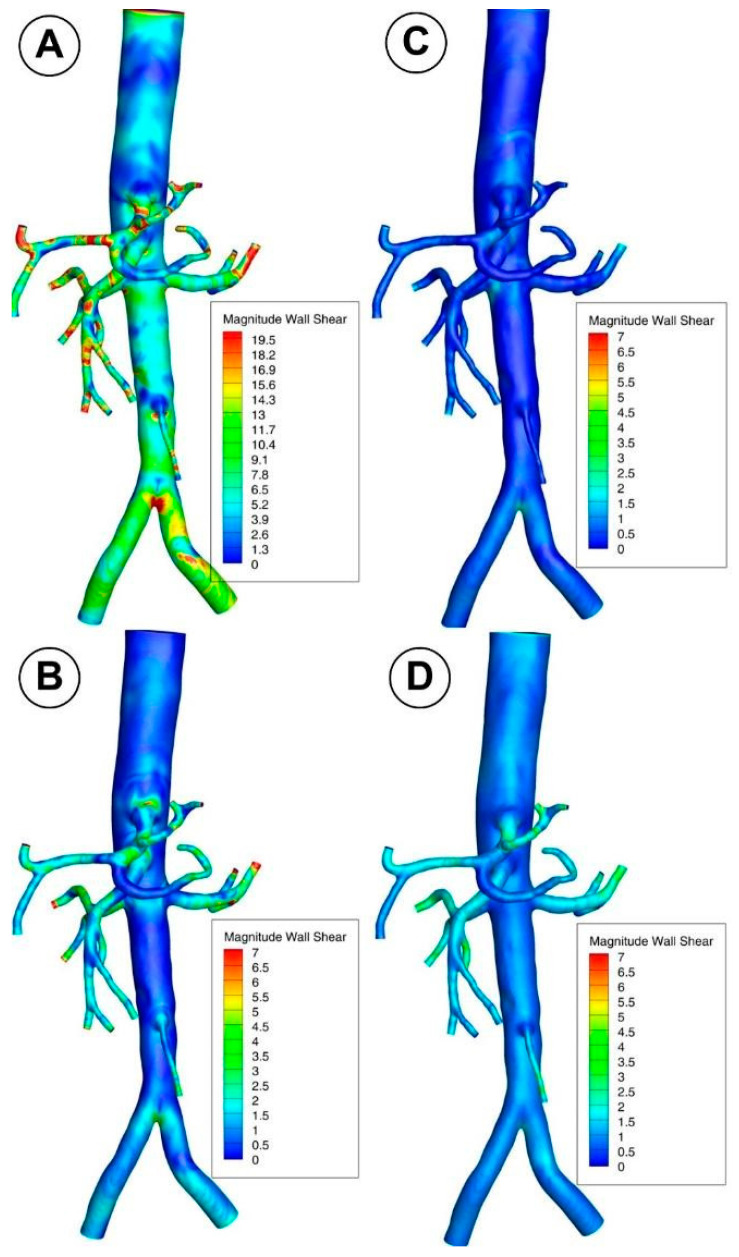
WSS distribution in the investigated abdominal aorta for different time steps. (**A**) T1 = 0.15 s; (**B**) T2 = 0.45 s; (**C**) T3 = 0.6 s; (**D**) T4 = 1 s.

A decrease in the inlet area of the artery reduces the WSS magnitude from the proximal to the middle segment for the abdominal aorta and each bifurcation, according to a positive correlation between the two variables. In the case of the abdominal aorta, branching arteries diminish flow rate, causing a decrease in WSS compared with the proximal segment before branching (Figure 12). As a result, it makes the middle segment more susceptible to forming atherosclerotic plaque as presented in our early work [27].

Furthermore, the subsequent abdominal aorta segment’s hemodynamic activity is influenced by the celiac artery (CA) branch (Figure 8; Figure 12). Therefore, the asymmetry of the velocity profiles in that area is caused by a CA branch. As a result, there would be a noticeable difference in the WSS between the inner and outer curvature regions. In essence, the asymmetry would cause one wall’s WSS to decrease and its WSS on the other side to increase. In the current study, the artery bifurcation region shows a more significant rise in disturbed flow than the regions of the branch curvature, creating favorable atherogenic conditions in this segment.

Lower WSS values are brought about by a reduction in abdominal aorta diameter in the distal segment (between the aortorenal bifurcation and inferior mesenteric artery branch). The magnitude of the WSS decreases from the proximal section of the abdominal aorta to the distal segment as the area decreases (distal to the aortorenal bifurcation region). Again, branching arteries lower the flow velocity through the distal abdominal aorta section, which may lead to a lower WSS than the proximal segment before branching. This mechanism brings on a tendency to hemodynamic behavior linked to plaque development (induces a more disturbed flow which is directly correlated with hemodynamic conditions that may lead to atherosclerotic plaque formation).

The local hemodynamic condition may impact the endothelial function, leading to intimal hyperplasia. In addition, branching arteries changed the distributions of WSS. The leading causes of neointimal development and local stenosis are a larger area and abrupt change in WSS between the bifurcation and the arterial branches, as well as the presence of recirculating and swirling flow [51,52].

## 4. Discussion

### 4.1. Velocity Field

**Figure 13:**
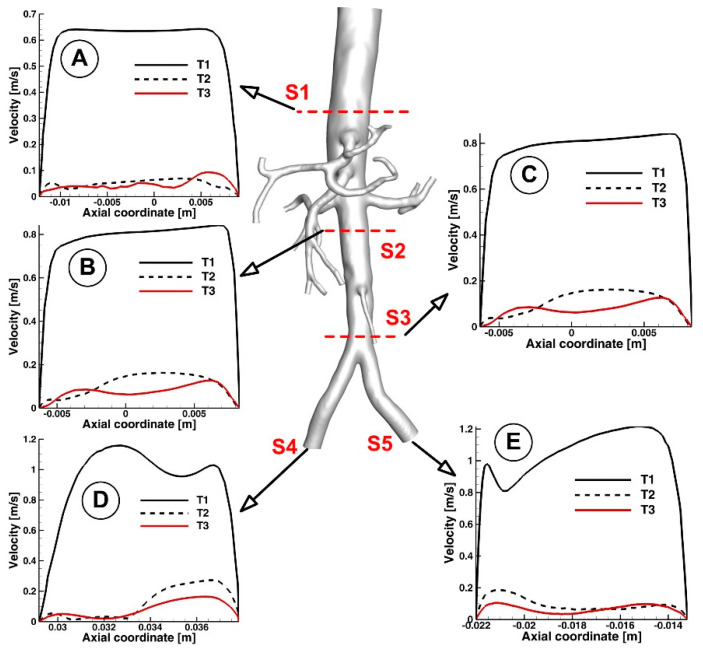
Cross-sectional flow pattern evolution in a different section of the investigated aorta. Velocity profile representation along the diameter associated to the cross-sections (**A**) S1, (**B**) S2, (**C**) S3, (**D**) S4 and (**E**) S5.

Cross-sectional views of the flow pattern may help study the characteristics of the flow field. For example, the flow of the examined abdominal aorta is shown in Figure 13 in several cross sections at various points.

The branching daughter vessels and the effect of the vessel’s curvature have been demonstrated to have a significant impact on the secondary flow [53]. The vessel’s inner wall is where the streamwise flow’s velocity is distorted (Figure 13). This finding is consistent with several other investigations [54], which also found skewed and reversed flow around the vessel’s inner wall.

Key characteristics of the velocity distribution included skewed velocity toward the inner walls of the iliac arteries (Figure 13D,E), the “M” shape profile in the exit sections of the LIA and RIA during the cardiac cycle, and velocity distribution in the investigated abdominal aorta’s proximal part (sections S1, S2, and S3).

**Figure 14:**
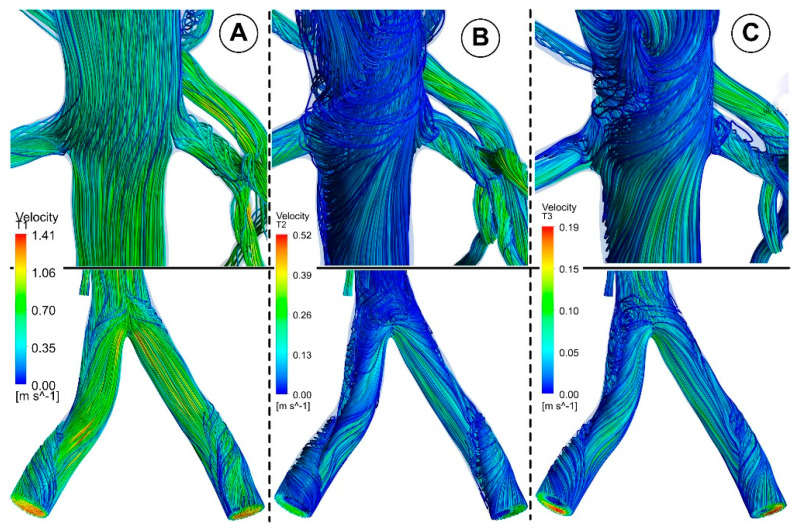
Recirculation development during different time steps (**A**) time T1 = 0.15 s; (**B**) time T2 = 0.45 s; (**C**) time T3 = 0.6 s in; renal arteries bifurcation (**upper row**) and iliac artery bifurcation (**lower row**).

The abdominal aorta model has two areas that are vulnerable to flow recirculations. The posterior wall of the aorta, close to the renal arteries, is the first and most noticeable (Figure 14). The area is highly likely to develop plaque, as demonstrated by clinical evidence. The recirculation size varies with the flow rate during a pulse cycle [26,41]. At the peak flow rate (time T1), it was observed that the zone was minimal (but not gone; see Figure 14A), and it then reappeared when the flow rate declined (Figure 14B,C upper row). Most of the time, the recirculation zone was present during the cardiac cycle. The experimental results of Moore and Ku [48], Mostbeck et al. [54], and this conclusion were mainly in agreement. In terms of fluid dynamics, a recirculation zone is a region of slow, fluctuating flow.

Near the exterior walls of the common iliac arteries is the second area for recirculation (Figure 14, lower row). When the flow rate decreased, it was observed that these places grew the greatest. The flow field exhibits a velocity profile skewed toward the inner walls in the x-y plane (Figure 13D,E). As a result, the sections next to the inner wall have low inertia, eventually developing a recirculation zone. These areas may experience plaque development clinically.

**Figure 15:**
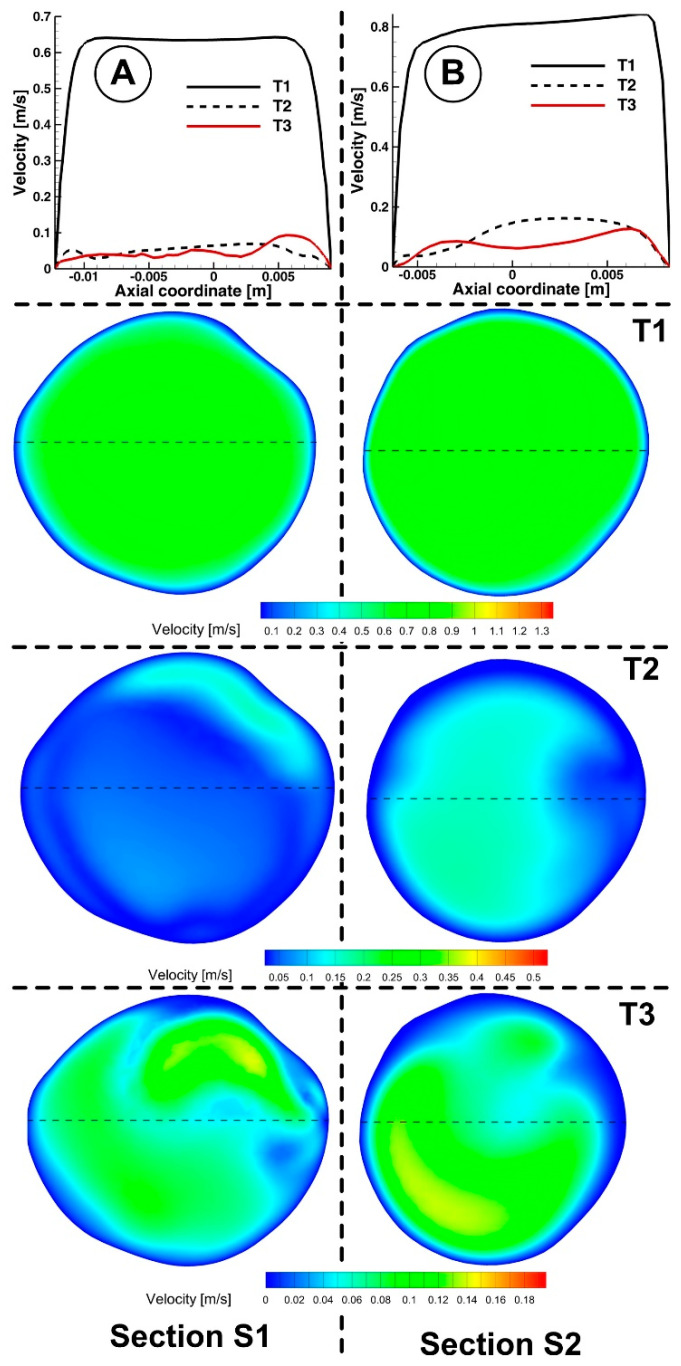
Velocity magnitude contour in different time steps in sections S1 (**A**) and S2 (**B**) (see Figure 13). The velocity evolution was plotted approximately along the section diameter (dashed line in each section).

The flow field is shown in Figure 15. Secondary motions can be seen in the cardiac cycle and the presence of arterial branches. The secondary motion was minimal in the core area. The recirculation zone, which corresponds to the time steps T2 and T3 from the cardiac cycle, could be observed on the outside wall in the cross-section of planes S1 and S2.

### 4.2. Wall Shear Stress Evolution

The vascular tree’s geometric structure includes straight, curved, branched, converged, diverging, and other complex traits, making the hemodynamic environment quite complex. Hemodynamic shear stress is continuously present on the blood vessel’s luminal and endothelial surfaces [55].

Endothelial cells’ shear-controlled gene expression was probably developed to keep vascular structural and functional homeostasis at a local level by transducing hemodynamic shear [56]. The atherosclerotic-protective endothelium phenotype appears to be produced by shear stress at physiological arterial magnitudes (>15 dyne/cm^2^) [57].

With average shear stress of 10 to 70 dyne/cm^2^ on the vascular ECs (endothelian cells), the hemodynamic flow pattern in the straight segment of an artery is typically laminar [58]. However, the hemodynamic flow becomes disturbed in the curved, branched, and diverging portions of the artery tree, causing eddies and low and reciprocating shear stress zones. As a result, the flow condition is known as irregular flow [59,60].

In contrast, due to vascular network architectural constraints (0–4 dyne/cm^2^), the outer walls of vessel bifurcations are characterized by modest and oscillatory shear stress and are susceptible to atherosclerosis [57]. In addition, these focused areas exhibit increased endothelial cell cycling and susceptibility to systemic apoptogenic stimuli, including tumor necrosis factor and oxidized low-density lipoprotein.

While our result shows that the shear stress on the outside lateral wall is relatively low, the peak shear stress at the flow divider is more significant than 100 dyne/cm^2^. This outcome is comparable to outcomes reported in Malek’s study [55].

**Figure 16:**
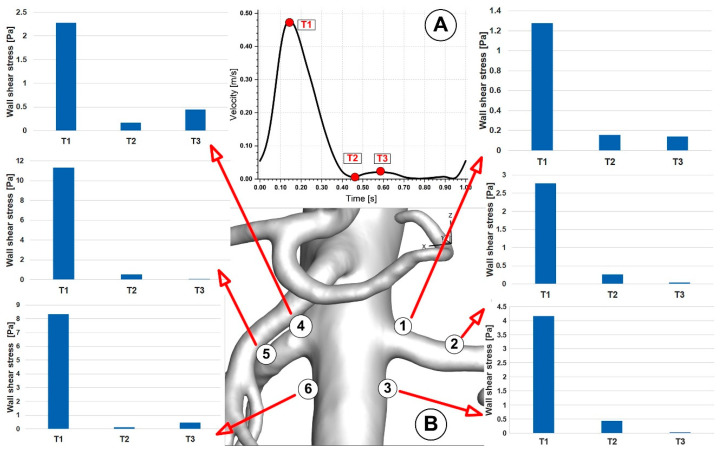
WSS evolution at various locations along the renal arteries (**B**) during different time steps (corresponding to time T1, T2, and T3, respectively (**A**)).

**Figure 17:**
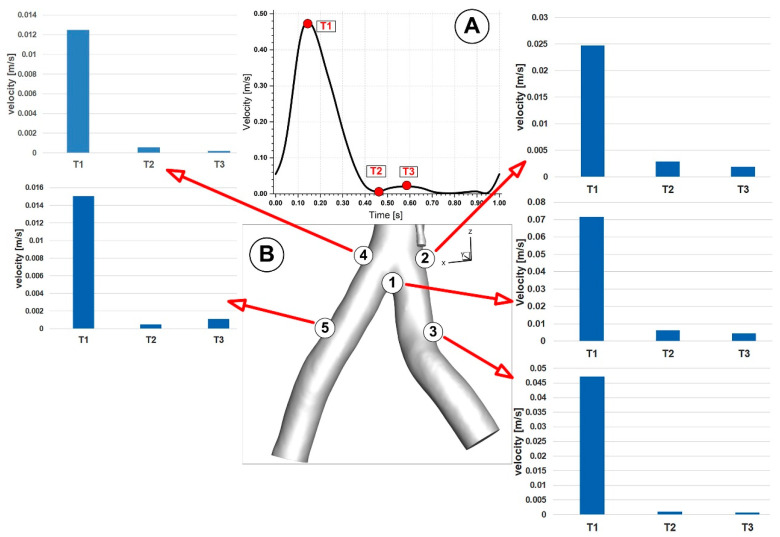
Velocity evolution at various locations along the iliac artery bifurcation (**B**) during different time steps (corresponding to time T1, T2, and T3, respectively (**A**)).

**Figure 18:**
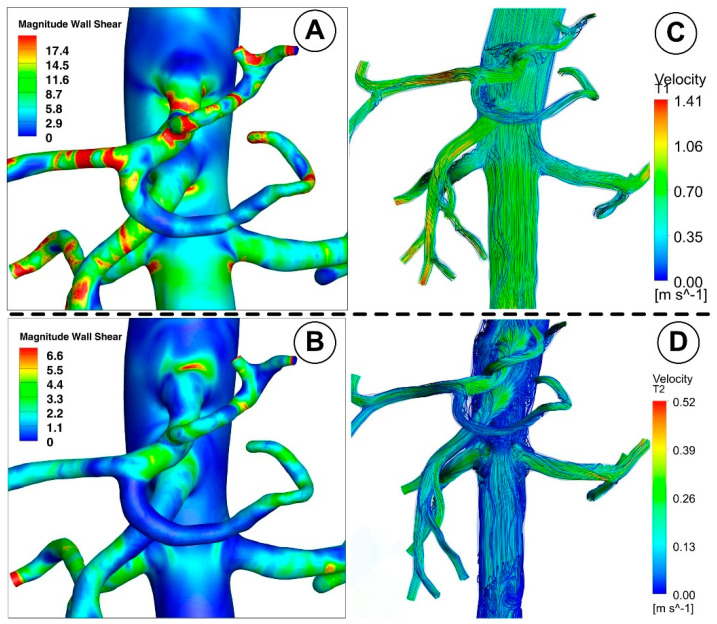
WSS contour in the renal arteries segment for different time steps (**A**) time T1, (**B**) time T2. The recirculation development is directly correlated with WSS evolution. Flow evolution is presented through path lines (**C**) time T1 and (**D**) time T2.

We looked at several points along the bifurcation wall to better understand the WSS evolution in arterial bifurcations. The distribution of WSS at various locations along the renal and iliac arteries throughout the cardiac cycle is shown in Figure 16 and Figure 17. In addition, Figure 18 shows the progression of WSS along the posterior wall in the section of renal branches at various time steps to help understand how WSS evolves during the cardiac cycle.

### 4.3. Clinical Relevance

This work shows how methods for precisely reproducing the patient-specific hemodynamics of complex abdominal aorta shapes can be created by combining in vitro and silico techniques. Well-informed clinical planning cannot rely solely on geometric information; it must also consider the pathological and complex hemodynamic environment that characterizes the evolution of complex diseases in the abdominal aorta and branches. Recent literature demonstrates the significant impact of fluid dynamic markers, such as WSS, on the long-term progression of atherosclerosis [61].

**Figure 19:**
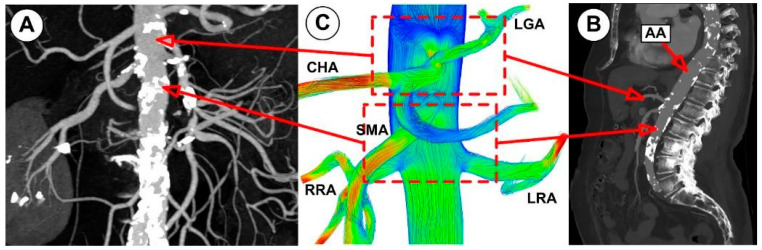
A CFD model was used to estimate the stenosis-prone area in the inferior aorta and its branches. (**A**) Anterior view of the inferior aorta segment with severe stenosis and calcification (patient A; (**B**) lateral view of the stenosed abdominal aorta (patient B); (**C**) flow field evolution predicted using numerical simulation in the region of the renal artery bifurcation. The CFD results (obtained for a healthy patient) can estimate the sites prone to stenosis development in different segments of the abdominal aorta.

**Figure 20:**
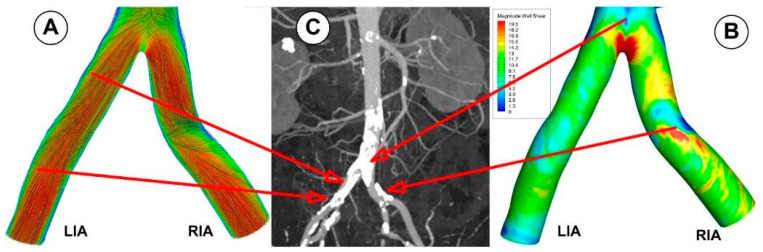
Correlation between WSS evolution obtained using numerical analysis (**A**,**B**), and disease location found during CTA investigation (**C**). CFD models estimate the hemodynamic markers (WSS) in the iliac artery bifurcation.

As seen in Figure 19 and Figure 20, CFD models can be used to estimate in detail hemodynamic markers like WSS that are impossible to assess in vivo and difficult to test experimentally. Additionally, in silico models can be employed to simulate various interventional settings (bypass grafting, stent employment, drug targeting).

The cardiac circulation, carotid artery, aorta, aorta branches, renal arteries, iliac bifurcation, and lower peripheral arteries are only a few of the arterial regions that have been widely discussed in the literature in the development of atherosclerosis [12,13,14]. Until recently, several clinical and imaging investigations emphasized the development of atherosclerosis in the locations mentioned above. However, the advancement of CFD over the past 20 years has made it possible to analyze some fundamental hemodynamic factors in detail, including WSS and the establishment and extension of recirculation zones [17,19,52], which are the foundation for the onset and progression of cardiovascular illnesses. These numerical studies also demonstrated their potential for planning therapeutic interventions and identifying arterial regions where vascular diseases are more likely to arise [26,44,62].

The ability of CFD to identify arterial regions at higher risk of later developing diseases is seen in Figure 19 and Figure 20. Therefore, the abdominal aorta section under study in the article’s numerical analysis for a patient without deposits on the vascular wall revealed the arterial segments that present a high risk for atherosclerosis from the perspective of the flow parameters. To show this, we contrasted the findings of three different patients’ CTA-type imaging investigations with the paper’s numerical findings. It is clear from a close examination of Figure 19; Figure 20 that the numerical findings accurately anticipate the locations of atheromatous plaque development in the middle segment of the aorta, the region of the renal branches, and the region of the iliac bifurcation and its branches. A crucial tool for enhancing the outcomes of studies and applied medical therapies is the capability provided by CFD analysis to explore fundamental hemodynamic parameters like the WSS and the recirculation areas (which clinical and imaging methods are unable to emphasize).

### 4.4. Limitation

The following suppositions are adjusted for the current methodology: (I) the artery wall is non-elastic; (II) cardiac motions are ignored. There is always the chance of modeling these presumptions, which results in more precise forecasts.

Regarding the rheology of the fluid used to simulate blood, a compromise had to be made in the current investigation. Therefore, our simulation used the Newtonian fluids as blood analog solutions. In the following stage, a simulation with the rheological characteristics of a non-Newtonian blood model (Carreau model [62]) will be carried out to assess the influence of these approximations.

We have shown in the current work how the flow transition influences clinically significant hemodynamic parameters, such as velocity and WSS. CFD can result in accurate quantitative studies and fluid flow events forecast when performed using a robust numerical method.

The lack of a more significant number of reconstructions that would make it easier to emphasize the potential to employ the CFD in the a priori evaluation of arterial areas prone to the development of vascular diseases is a significant drawback of the article. It is well-recognized that every patient has a distinct anatomical structure, and the circulatory system is no exception. High arterial tortuosity, unusual 3D orientation, abnormal arterial origin, and abnormal arterial diameters are crucial elements that alter the configuration of blood flow in arterial segments. The following inquiries must be addressed through CFD investigations:

Certification of the accuracy of numerical results in comparison to the findings of medical investigations (MRI, CTA, and PET-SCAN).

Calibration of reconstruction techniques and numerical analyses to obtain results following the actual situation of the patient, regardless of their anatomical and pathological particularities.

The possibility of performing routing (to include the CFD investigation in the medical protocols of investigation and planning of treatments).

In the following steps, we aim to conduct CFD investigations for many patients, allowing us to formulate an algorithm or strategy with high precision regarding the degree of susceptibility of the various arterial segments depending on the individual hemodynamic regime of each patient.

## 5. Conclusions

A patient-specific hemodynamic analysis model was used in this study to simulate the blood flow dynamics in the abdominal aorta and its branches by integrating in vivo CT imaging with in silico simulation under healthy conditions. The findings showed that complex flow predominates at the site of arterial bifurcations, where aberrant flow patterns produce recirculation zones with low and fluctuating WSS (0.5 Pa), which are known to impair endothelial homeostasis and result in adverse vascular remodeling. In addition, the range changes of critical hemodynamic variables, such as WSS and recirculation region enlargement, closely related to the initiation and development of atherosclerosis, were quantified.

A better knowledge of blood flow in the abdominal aorta and its branches under physiological settings was made possible by including in vivo images in the computational model. According to the realistic model geometry, the recirculation area occurs preferentially downstream of the bifurcation at the outer walls. The findings also show that the cardiac cycle and its fleeting nature significantly impact atherosclerosis, with the enlargement of the recirculation zone playing a more significant role during the deceleration phase.

The simulations based on a realistic model gave extensive information, both qualitatively and numerically, concerning the blood flow in the examined area. These studies are templates for additional hemodynamic research that can be employed as prediction tools to direct patient management, from clinical diagnosis to individualized therapy plans [63]. Furthermore, it should be emphasized that these models provide a promising method to research nanoparticle-mediated medication targeting the treatment of atherosclerosis by providing a better understanding of blood flow patterns at this vascular level [64,65]. A promising area of study that hopes to establish a new, non-invasive computational paradigm for medical diagnosis, pretreatment planning, and individualized therapy has also made recent strides in integrating CFD with deep learning [66].

## Figures and Tables

**Table 1:** Reconstructed arteries’ geometrical parameters.

Artery	Diameter Measured in the Paper [mm]	Diameter Mentioned in References [mm]	References
Aorta (inlet section)	22.00	14–30	[29]
Celiac artery	7.16	8.57 ± 1.57	[30]
Superior mesenteric artery	7.84	8.35 ± 1.60	[30]
Left renal artery	7.12	7.03 ± 1.40	[30]
Right renal artery	7.00	7.13 ± 1.20	[30]
Inferior mesenteric artery	4.04	4.21 ± 1.20	[30]
Left iliac artery	11.00	11.77 ± 2.20	[30]
Right iliac artery	10.90	12.23 ± 2.40	[30]

**Table 2:** Different mesh configurations are used for the mesh sensitivity test.

Mesh	Number of Nodes (Total)	Number of Elements (Total)	Number of the Nodes Inlet Section	Number of Elements (Inlet Section)
Coarse (mesh#1)	1,011,403	2,885,022	3156	4582
Medium (mesh#2)	1,951,507	6,566,740	4154	6386
Fine (mesh#3)	3,479,629	9,558,980	8072	13,784

**Table 3:** Flow distribution in the abdominal aorta and the side branches.

Cross-Section	Flow Rate [L/min]	Percentage Distribution [%]
Inlet	5.644	100
o1	0.043	0.76
o2	0.071	1.25
o3	0.076	1.35
o4	0.153	2.72
o5	0.092	1.63
o6	0.101	1.78
o7	0.077	1.37
o8	0.057	1.01
o9	0.133	2.35
o10	0.175	3.10
o11	0.102	1.82
o12	0.063	1.11
o13	0.237	4.20
o14	0.045	0.80
o15	2.228	39.48
o16	1.991	35.28

For a definition of the cross-section, see Figure 8.
